# Development and Application of an Intelligent Diagnosis System for Retinal Vein Occlusion Based on Deep Learning

**DOI:** 10.1155/2022/4988256

**Published:** 2022-08-24

**Authors:** Wei Xu, Zhipeng Yan, Nan Chen, Yuxin Luo, Yuke Ji, Minli Wang, Zhe Zhang

**Affiliations:** ^1^Department of Optometry, Jinling Institute of Technology, Nanjing, Jiangsu, China; ^2^Nanjing Key Laboratory of Optometric Materials and Application Technology, Nanjing, Jiangsu, China; ^3^The Laboratory of Artificial Intelligence and Big Data in Ophthalmology, Affiliated Eye Hospital of Nanjing Medical University, Nanjing, Jiangsu, China; ^4^The First Affiliated Hospital of Huzhou University, Huzhou, Zhejiang, China; ^5^Shenzhen Eye Hospital, Jinan University, Shenzhen, Guangdong, China

## Abstract

This study is aimed at developing an intelligent algorithm based on deep learning and discussing its application for the classification and diagnosis of retinal vein occlusions (RVO) using fundus images. A total of 501 fundus images of healthy eyes and patients with RVO were used for model training and testing to investigate an intelligent diagnosis system. The images were first classified into four categories by fundus disease specialists: (i) healthy fundus (group 0), (ii) branch RVO (BRVO) (group 1), (iii) central RVO (CRVO) (group 2), and (iv) macular branch RVO (MBRVO) (group 3), before being diagnosed using the ResNet18 network model. Intelligent diagnoses were compared with clinical diagnoses. The specificity of the intelligent diagnosis system under each attention mechanism was 100% in group 0 and also revealed a high sensitivity of over 95%, *F*1 score of over 97%, and an accuracy of over 97% in this group. For the other three groups, the specificities of diagnosis ranged from 0.45 to 0.91 with different attention mechanisms, in which the ResNet18+coordinate attention (CA) model had the highest specificities of 0.91, 0.88, and 0.83 for groups 1, 2, and 3, respectively. It also provided a high accuracy of over 94% with a coordinate attention mechanism in all four groups. The intelligent diagnosis and classifier system developed herein based on deep learning can determine the presence of RVO and classify disease according to the site of occlusion. This proposed system is expected to provide a new tool for RVO diagnosis and screening and will help solve the current challenges due to the shortage of medical resources.

## 1. Introduction

Retinal vein occlusion (RVO) is a common retinal vascular degenerative disease with an increasing prevalence in individuals aged 30–89 years, which makes the blindness rate higher than that of other retinal vascular disorders besides diabetic retinopathy [1]. Characterized by retinal vein filling, proximal vascular occlusion, distal vascular dilation, retinal hemorrhage, and edema due to ischemia and hypoxia, possible causes of RVO include external compression or disease of the vein wall, such as vasculitis [2, 3]. Based on the location of the blocked blood vessels, RVO can be divided into two primary categories depending on the site of occlusion: branch RVO (BRVO) and central RVO (CRVO) [4]. Sight-threatening complications of RVO include macular edema, macular ischemia, and vitreous hemorrhage due to retinal neovascularization. This damages the visual function of patients and even causes permanent and irreversible vision loss [5–7].

The treatment of RVO mainly focuses on its etiology including hypertension, arteriosclerosis, and inflammation and complications, such as macular edema, ischemia, and neovascularization [8]. Currently, intravitreal therapy is an economical and effective method for the treatment of this disease [9]. In addition, laser photocoagulation has been recommended for patients with neovascularization and macular edema, and many surgical treatment modalities have been reported for critical patients [8, 10]. The choice of treatment should be personalized to the individual patient for different subtypes of RVO; therefore, diagnosis and differential diagnosis are important. Fundus examination and fundus photography are important methods for the preliminary evaluation of RVO. Other imaging diagnostic methods, such as fluorescein angiography (FA) and optical coherence tomography (OCT), are also widely used for detection and evaluation [11].

With the wide application of deep learning in the field of image recognition, an increasing number of ophthalmologists and intelligent technologists have begun to explore image recognition and classification technologies based on deep learning and apply it to the clinical diagnosis and treatment of retinal diseases. Chandrakumar et al. [12] classified fundus images using a 13-layer convolutional neural network. Ardiyanto et al. [13] proposed a compact deep learning algorithm with small, embedded plates for detecting diabetic retinopathy. Kermany et al. [14] established a classifier system for screening age-related macular degeneration (AMD) and diabetic macular edema, utilizing transfer learning on a dataset of OCT images. Li et al. [15] proposed an OCT image segmentation algorithm based on a 3D neural network to solve the problem of retinal fluid segmentation. Wan [16] presented a convolutional neural network named EAD-Net that can achieve pixel-level accuracy for different types of lesions in diabetic retinopathy. Xu et al. [17] proposed two biomarker segmentation schemes integrating the semiautomatic localization technique and the low-rank and sparse decomposition theory to locate the leakage area in laser surgery of chronic central serous chorioretinopathy. Promoting the deep integration of artificial intelligence (AI) and medical care will help alleviate problems due to shortage of specialized medical resources in China and will improve the efficiency of disease screening. This study is aimed at developing an intelligent algorithm based on deep learning and discussing its application for the classification and diagnosis of RVO using fundus images, expected to aid in promoting the early diagnosis and treatment of treatable RVO cases, thereby achieving better prognosis.

## 2. Materials and Methods

### 2.1. Ethical Approval

This study was approved by the Institutional Research Ethics Committee of Nanjing Medical University and followed the tenets of the Declaration of Helsinki. All fundus photographs were anonymized before inclusion and contained no information of the patients, except for the diagnoses.

### 2.2. Image Acquisition and Preprocessing

The dataset used in this study was acquired from the Eye Hospital affiliated with Nanjing Medical University and contained 501 fundus images. All photographs were taken using a nonmydriatic fundus camera over 45° of the posterior pole. The fundus images were complied with Chinese annotation and quality control specifications for fundus colour photographs [18], and data anonymization was applied before the study.

**Figure 1:**
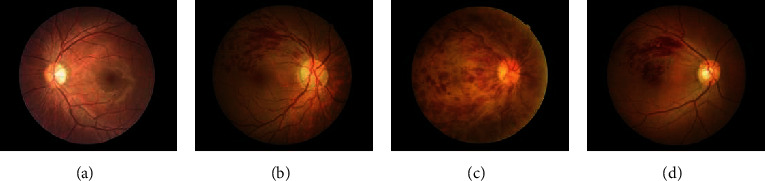
Four categories of the fundus images. (a) Group 0: healthy fundus. (b) Group 1: branch retinal vein occlusion (BRVO). (c) Group 2: central retinal vein occlusion (CRVO). (d) Group 3: macular branch retinal vein occlusion (MBRVO).

The images were classified into four categories by fundus disease specialists as follows: (i) healthy fundus (group 0), (ii) BRVO (group 1), (iii) central CRVO (group 2), and (iv) MBRVO (group 3) (as shown in Figure 1). Then, they were randomly allocated into the test, training, and validation sets. The samples of the training set and the other two sets were divided in a ratio of 2 : 8 (Table 1).

The dataset covering 501 photographs was not adequate for the deep convolutional neural network in this study; therefore, it was augmented to decrease the phenomenon of overfitting. Only the original data were appropriately transformed without changing the amount of original data or introducing any irrelevant data to increase the sample data and improve the generalizability of the model. Data augmentation was applied only to the training set in an online form, which was omitted from the statistics. The methods of adjustment included image inversion, image rotation, image compression, image random cropping, brightness adjustment, and gamma correction.

### 2.3. Model Training and Evaluation

**Figure 2:**
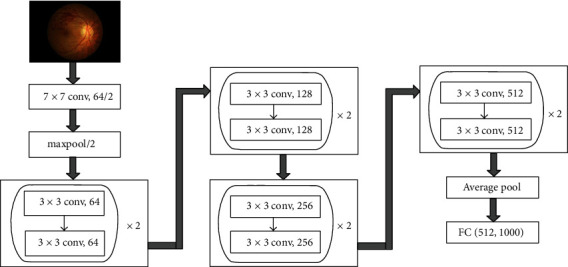
Architectural diagram of ResNet18.

The main network framework used in this study was a convolutional neural network called ResNet18, which is a fusion of the inception and residual networks. The basic architecture of the ResNet18 model includes convolutional, max pooling, and activation layers and a fully connected layer (Figure 2). The batch size of the model was four per card, and the gradual warmup method was used for learning rate optimization. A total of 50 epochs were trained, with a learning rate set between 10^−2^ and 10^−5^. The best learning rate was selected by comparing the output results for different learning rates. One of the three attention mechanisms, coordinate attention (CA), convolutional block attention module (CBAM), and squeeze and excitation network (SENet), was added to the basic network model of ResNet18 for the controlled study. Networks with different attention mechanisms were trained on the same dataset and treated them in the same way.

Intelligent diagnosis was performed based on fundus images. The same images were consulted by three retinal specialists in a double-blind trial. Final clinical diagnostic results were created for two or more identical grading diagnoses, taken as the expert diagnosis. The sensitivity, specificity, *F*1 score, and accuracy of the intelligent diagnosis system were calculated by comparing the results of expert and intelligent diagnoses.

### 2.4. Statistical Analysis

Statistical analyses were conducted by SPSS 24.0. Enumeration data were represented by the number of images and indicators including accuracy, specificity, sensitivity, and *F*1 score.

## 3. Results

In this study, 501 fundus images were used to evaluate the proposed intelligent grading and diagnosis system for RVO. According to the analysis of the above results, the training effect of the model achieved the best results, with a learning rate of 10^−5^. Therefore, the learning rate was set at 10^−5^ and the other parameters remained unchanged. The training results of different networks were compared.

Compared with the expert diagnosis, the positive predictive value (PPV) and specificity of the intelligent diagnosis system under each attention mechanism were almost 100% in group 0. The method also provided a high sensitivity of over 95% and an accuracy of over 97% in this group. For the other three groups, the specificities of diagnosis differed from 0.45 to 0.91 with different attention mechanisms, in which the ResNet18+CA model had the highest specificities of 0.91, 0.88, and 0.83 in groups 1, 2, and 3, respectively. The highest sensitivities for the intelligent diagnosis were 100% in group 0 and 100% in group 2. Overall, the intelligent diagnosis system provided a high accuracy of over 94% with a coordinate attention mechanism in all four groups (Table 2).

## 4. Discussion

The aim of this study was to investigate the possibility of constructing an intelligent diagnosis of RVO from fundus photographs using deep learning-based algorithms. Owing to the lack of fundus specialists, screening for RVO is difficult in areas lacking medical resources. The results of this study show that the deep learning model has high specificity, sensitivity, and accuracy for RVO detection and diagnosis. It also enables mass screening for fundus diseases in remote and rural areas.

Anitha et al. [19] evaluated 420 abnormal retinal images from four different categories (nonproliferative diabetic retinopathy (DR), CRVO, central serous retinopathy, and central neovascularization membrane). The authors used 95 images from CRVO for image classification using an identification technique for abnormal fundus images based on the Kohonen neural network (KNN). It showed a sensitivity of 0.97, specificity of 0.99, and an accuracy of 98% for CRVO detection. Zheng et al. [20] proposed a five-category intelligent auxiliary diagnosis model for common fundus diseases, including RVO, high myopia, glaucoma, and DR. The evaluation indicators of sensitivity, specificity, and *F*1 score were 88.27%, 95.99%, and 83.14%, respectively, in the RVO group. Overall, these results indicate that AI can identify RVO efficiently based on their appearance.

Previous studies have focused on RVO. Nagasato et al. [21, 22] created a deep convolutional neural network (DNN) model and trained it using preprocessed image data of CRVO and BRVO cases and that of non-RVO samples. The findings suggested that the proposed DNN model may be useful in diagnosing RVO by identifying suspected retinal hemorrhages, and the deep learning model has higher sensitivity, specificity, and AUC values than the support vector machine model for detecting RVO in fundus photographs. Thus, the proposed intelligent technique can aid in accurate diagnosis based on fundus images without human input and can be used for RVO screening and early diagnosis at a low cost for a large number of patients.

**Figure 3:**
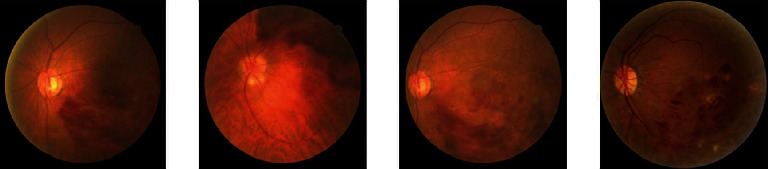
Examples of group 1 (BRVO).

**Figure 4:**
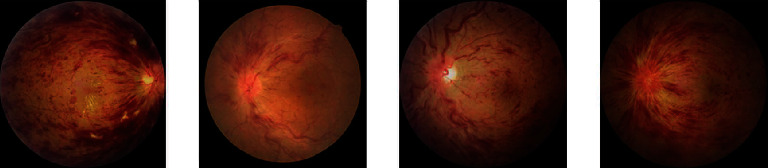
Examples of group 2 (CRVO).

**Figure 5:**
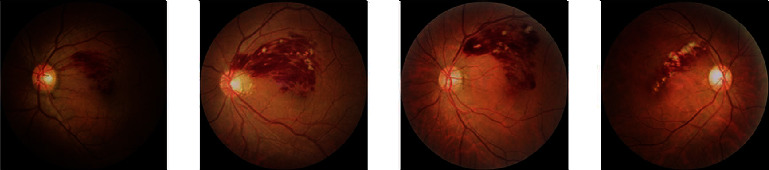
Examples of group 3 (MBRVO).

**Figure 6:**
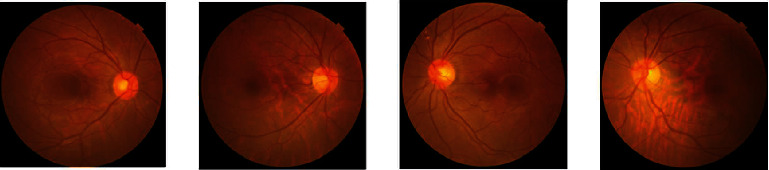
Examples of group 0 (healthy fundus).

In this study, the network performance improved when the attention mechanism was added, among which the ResNet18+CA achieved the best performance. The classification effect of the different networks for group 0 was better than that of the other three groups. The possible reason was that group 0, as healthy fundus images, accounted for the largest percentage in the whole dataset, and the number of the other three groups, including CRVO, BRVO, and MBRVO, shares an uneven proportion. In addition, the quality of the fundus images of different RVO types in the dataset varied greatly. The fundus images of BRVO, CRVO, and MBRVO showed significant differences in size, shape, brightness, and other aspects (Figures 3–5), whereas the healthy fundus images showed minimal difference in these aspects and were better in quality than the other three types (Figure 6). Therefore, the training results for the fundus images of group 0 were better than those for the other three groups. Similar findings were reported by Chen et al. [23].

Despite the above findings, this study had a few limitations. As suggested in the results, the intelligent diagnosis was less sensitive in groups 1 to 3 than in group 0, indicating further optimization and investigation of the diagnostic model. In future studies, the samples for model training should be enlarged, and the quality of the images should be controlled, as these elements are significant for model training and testing. With the development of artificial intelligence in ophthalmology, eye care practitioners have a better understanding of intelligent diagnosis than other professional technicians [24]. We expect more cooperation between ophthalmologists and experts in the field of artificial intelligence in attempt to define a more concise network model to reduce operating costs. Proceeding to the next stages, we consider comparing the accuracy of RVO diagnosis system with ophthalmologists of different stage and incorporating research on single RVO disease in multiple fundus diseases, to make the intelligent diagnosis system a better landing in clinic. Due to the lack of specialized ophthalmologists in remote or rural areas, screening for fundus diseases such as RVO still faces a huge gap. We expected to help promote the integration of artificial intelligence and healthcare and to solve the current medical resource shortage in the “last kilometer” in China.

## 5. Conclusions

This proposed system is expected to provide a new tool for RVO diagnosis and screening and will help solve the current challenges due to the shortage of medical resources.

This study focused on expanding the core theoretical models and key techniques required in the intelligent diagnosis for RVO. Based on deep learning algorithm, the intelligent diagnosis and classifier system developed herein can determine the presence of RVO and preferably classify disease according to the site of occlusion, and the intelligent diagnosis achieved was highly consistent with clinical diagnosis in all groups with the addition of the ResNet18+CA model.

## Figures and Tables

**Table 1:** Dataset.

*n*	Test set	Training set	Validation set	Total
Group 0 (healthy fundus)	62	166	31	259
Group 1 (BRVO)	24	76	17	117
Group 2 (CRVO)	14	45	10	69
Group 3 (MBRVO)	12	36	8	56
Total	112	323	66	501

**Table 2:** Evaluation index results of different models.

Model	Group				
	Evaluation indicators	Group 0	Group 1	Group 2	Group 3
ResNet18	Specificity	1.0000	0.5333	0.5556	0.5000
	Sensitivity	0.9516	0.6667	0.3517	0.5833
	*F*1 score	0.9752	0.5926	0.4348	0.5385
	Accuracy	0.9732	0.8036	0.8839	0.8929

ResNet18+SE	Specificity	1.0000	0.6500	0.6923	0.4500
	Sensitivity	0.9516	0.5417	0.6429	0.7500
	*F*1 score	0.9752	0.5909	0.6667	0.5625
	Accuracy	0.9732	0.8393	0.9196	0.8750

ResNet18+CBAM	Specificity	1.0000	0.6800	0.6429	0.6667
	Sensitivity	0.9839	0.7083	0.6429	0.6667
	*F*1 score	0.9919	0.6939	0.6429	0.6667
	Accuracy	0.9911	0.8661	0.9107	0.9286

ResNet18+CA	Specificity	1.0000	0.9091	0.8750	0.8333
	Sensitivity	1.0000	0.8333	1.0000	0.8333
	*F*1 score	1.0000	0.8696	0.9333	0.8333
	Accuracy	1.0000	0.9464	0.9821	0.9643

## Data Availability

The raw data supporting the conclusions of this article will be made available by the authors, without undue reservation.
